# Crohn's disease after multiple doses of rituximab treatment in a child with refractory nephrotic syndrome and an *ATG2A* mutation: a case report

**DOI:** 10.3389/fped.2024.1464757

**Published:** 2024-11-20

**Authors:** Kaili Shi, Mengzhen Fu, Wei Xia, Pei Zhang, Chunlin Gao, Zhengkun Xia

**Affiliations:** ^1^ Department of Pediatrics, Jinling School of Clinical Medicine, Nanjing Medical University, Nanjing, Jiangsu, China; ^2^ Department of Pediatric Nephrology, BenQ Medical Center, The Affiliated BenQ Hospital of Nanjing Medical University, Nanjing, Jiangsu, China; ^3^ Department of Pediatrics, Jinling Hospital Affiliated with Nanjing University Medical College, Nanjing, Jiangsu, China

**Keywords:** rituximab, refractory nephrotic syndrome, inflammatory bowel disease, Crohn's disease, *ATG2A*

## Abstract

*De novo* inflammatory bowel disease (IBD) in response to rituximab (RTX) has been documented on multiple occasions as a severe adverse effect. However, none of these reports mentioned any genetic variation associated with this complication. We describe the case of a 16-year-old patient with refractory nephrotic syndrome (NS) diagnosed at the age of 6 years, notably with a heterozygous mutation of the *ATG2A* gene, who developed Crohn's disease (CD) following ten administrations of RTX. Seventy months after the first and 6 months after the last RTX dose, the patient developed recurrent abdominal pain, hematochezia, oral aphthous ulcers and weight loss. On the basis of clinical evaluation and ileo-colonoscopy findings, the patient was diagnosed with CD and treated with mesalazine. A significant amelioration of clinical symptoms was achieved after 11 days of mesalazine treatment. A repeat ileo-colonoscopy performed 4 months later revealed near-complete resolution of the ulcers and marked mucosal healing. The underlying pathophysiology of RTX-induced IBD has not yet been clarified. Autophagy associated with *ATG2A* mutation is likely involved in the pathogenesis. This case underscores the need for vigilance in monitoring children with NS with gastrointestinal symptoms following RTX treatment, especially those who have hereditary susceptibility and have received multiple administrations.

## Introduction

Rituximab(RTX) has been widely used to treat childhood idiopathic nephrotic syndrome (NS). In general, RTX appears to be safe for most children (1, 2). *De novo* RTX-induced inflammatory bowel disease (IBD) is a rare adverse event. We report the case of a 16-year-old patient with NS and an *ATG2A* mutation who developed Crohn's disease (CD) following multiple administrations of RTX. Symptoms at presentation included recurrent abdominal pain, hematochezia, oral aphthous ulcers and weight loss. Ileo-colonoscopy showed multiple, segmental and longitudinal ulcerations in the colon, and intestinal skip lesions. The patient was ultimately diagnosed with CD and treated with mesalazine, which achieved a positive effect. Mutation of the *ATG2A* gene led the patient to be susceptible to RTX-induced IBD. We believe that the present report is the first case report on RTX-induced IBD in a patient with an *ATG2A* mutation.

## Case report

A 16-year-old patient previously diagnosed with refractory NS visited the clinic for recurrent abdominal pain and hematochezia. His medical history revealed that he was first diagnosed with NS at the age of 6 years, and remission was achieved within a month of oral steroid therapy initiation. However, the patient's NS frequently relapsed on high doses of steroids in combination with mycophenolate mofetil (MMF), cyclophosphamide (CTX), or calcineurin inhibitors (CNIs). A kidney biopsy was performed at age 7 years, which revealed the tip variant of focal segmental glomerulosclerosis (Supplementary Figure 1). Medical Whole Exome Sequencing (mWES) revealed a heterozygous mutation in the *LMX1B* gene (chr9:129376748, c.20C>G, p.7, P>R) inherited from the patient's mother in an autosomal dominant manner. This mutation is classified as a missense mutation. Structural predictions indicate that this alteration is detrimental to protein function. Notably, the same mutation in the *LMX1B* gene has previously been reported in a patient with NS, and case reports support an association between SSNS and *LMX1B* mutations (3–5).

Considering his frequent replase of NS despite sequential treatments with MMF, cyclosporine A (CsA), CTX, tacrolimus, and prednisolone, he received one dose of RTX (375 mg/m^2^) at the age of 10 years. When the patient met the criteria for B-cell reconstitution (>1% of the total lymphocytes), he was treated with RTX again, even in the absence of NS relapses (6). Over a 5-year period, the patient received a total of ten RTX treatments and was found to have an improved response overall, with a longer relapse-free period (Supplementary Figure 2). Prednisolone and tacrolimus were tapered off to low doses. Notably, the patient was treated with recombinant human growth hormone (rHGH) because of growth hormone deficiency (GHD) at the age of 13 years.

Seventy months after the first and 6 months after the last RTX dose, the patient experienced back pain and then experienced transient low-grade fever and recurring abdominal pain with diarrhea. He was initially diagnosed with acute gastroenteritis. However, antibiotic treatments were ineffective. In rapid sequence, the patient developed hematochezia, watery stools four or five times daily, multiple oral ulcers and a weight loss of 3.5 kg. A physical examination of the patient revealed no notable abdominal mass or any discernible perianal lesions. The laboratory data at admission, reported in Table 1, were indicative of an inflammatory reaction, with an increasing white blood cell (WBC) count, C-reactive protein (CRP) level, and serum amyloid A (SAA) level. His erythrocyte sedimentation rate was elevated, and both complement C3 and C4 levels were above the normal range. The result of the infectious enteritis test was negative.

**Table 1:** Laboratory characteristics.

		Reference range			Reference range
Complete blood count			Biochemistry		
WBCs, 10^9^/L	11.90	4.10–11.00	TP, g/L	60.1	64.0–87.0
Neutrophils, 10^9^/L	7.36	1.80–8.30	ALB, g/L	33.9	35.0–50.0
NEUT%	0.619	0.370–0.770	TBIL, µmol/L	8.9	3.0–22.0
RBCs, 10^9^/L	4.37	4.50–5.90	ALT, U/L	13	1–40
Hb, g/L	117	129–172	AST, U/L	16	1–37
Ht	0.351	0.390–0.510	LDH, U/L	153	114–240
MCV, fL	80.3	80.0–100.0	ALP, U/L	113	0–110
MCHC, g/L	333.0	310.0–355.0	UA, µmol/L	366	200–430
MCH, pg	26.80	25.00–34.00	BUN, mmol/L	2.0	2.9–6.0
Platelets, 10^9^/L	410	150–407	CREA, µmol/L	52	53–115
Urinalysis			Other laboratory tests		
pH	7.0	5.4–8.4	CRP, mg/L	49.2	0.00–3.00
Specific gravity	1.008	1.003–1.030	SAA, mg/L	481.0	0.00–6.40
Protein	Negative		ESR, mm/h	43	≤20
Leukocytes, cells/ul	2	≤12	ANA	Negative	
Erythrocytes, cells/ul	4	<10	ANCA	Negative	
Stool examinations			C3, g/L	1.25	0.79–1.17
Fecal culture	Negative		C4, g/L	0.37	0.17–0.31
Entamoeba histolytica	Negative		IgA, g/L	3.54	1.45–3.45
Clostridium difficile Ag	Negative		IgG, g/L	9.41	10.13–15.13
RBC, WBC	Negative		IgM, g/L	0.42	0.92–2.04
Fecal hemoglobin	Positive		25(OH)D	31	>25 ng/ml
Fecal parasites	Negative		^13^C-urea breath test	Negative	
Toxigenic clostridium difficile	Negative		Serum CMV-IgM	Negative	
Genetic test			Tuberculosis PCR	Negative	
*HLA-B27* gene test	Negative		EB-VCA-IgG, U/ml	98.20	0.00–20.00
*NUDT15* gene test	Negative		EB-VCA-IgM, U/ml	0.50	0.00–40.00

WBCs, white blood cells; RBCs, red blood cells; NEUT%, percentage of neutrophils; Hb, hemoglobin, Ht, hematocrit; MCV, mean corpuscular volume; MCHC, mean corpuscular hemoglobin concentration; MCH, mean corpuscular hemoglobin; TP, total protein; ALB, albumin; TBIL, total bilirubin; ALT, alanine aminotransferase; AST, aspartate aminotransferase; LDH, lactate dehydrogenase; ALP, alkaline phosphatase; UA, uric acid; BUN, blood urea nitrogen; CREA, creatinine; CRP- C-reactive protein; SAA, serum amyloid A; ESR, erythrocyte sedimentation rate; ANA, antinuclear antibody; ANCA, anti-neutrophil cytoplasmic antibody; C3 and C4, complement 3 and 4, respectively; IgA, IgG, and IgM, immunoglobulin A, G, and M, respectively; 25(OH)D, 25-hydroxyvitamin D; CMV, cytomegalovirus; PCR, polymerase chain reaction; EB-VCA, Epstein–Barr virus viral capsid antigen.

**Figure 1:**
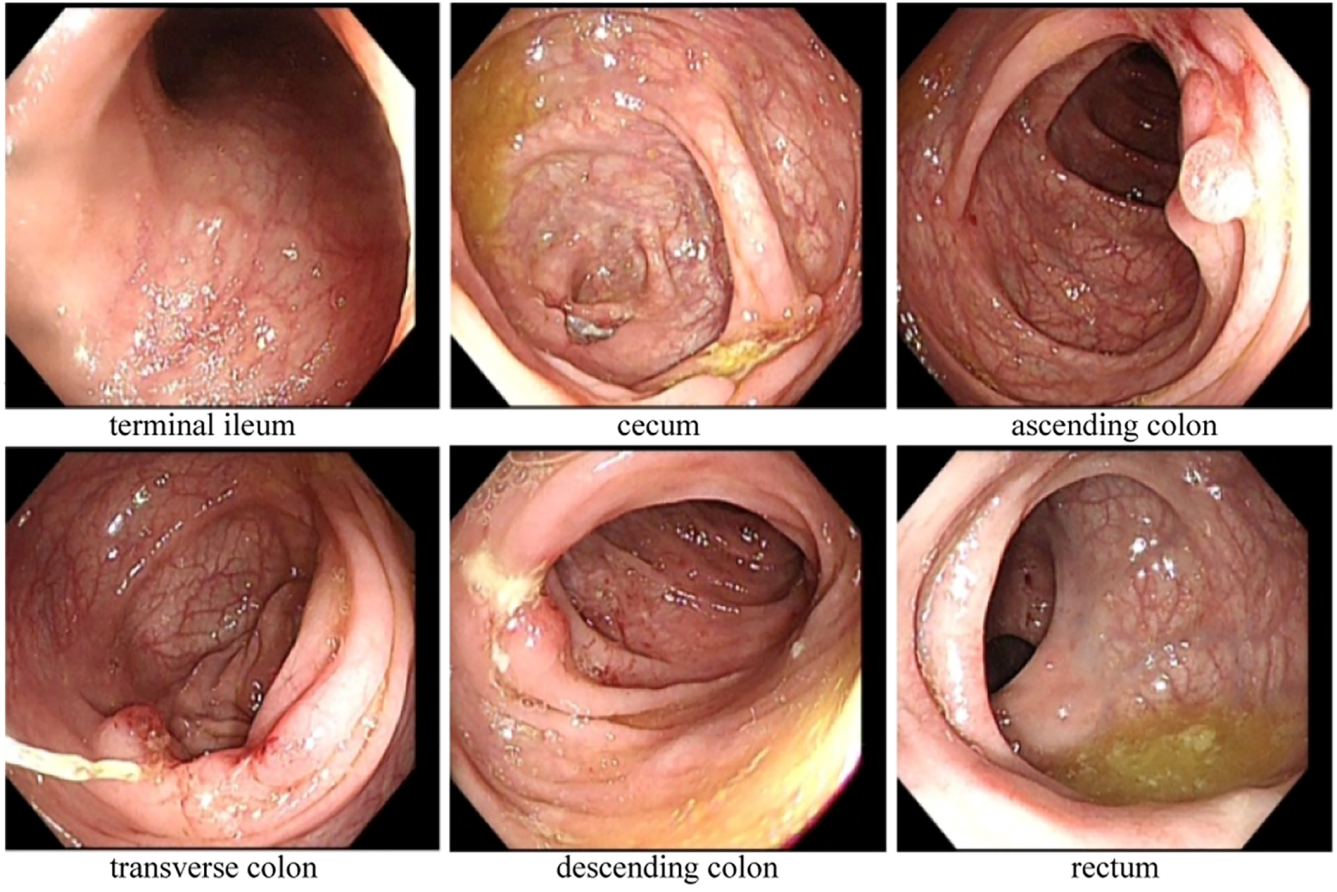
Ile-ocolonoscopy revealed that irregular ulcers of approximately 1 cm–2 cm in size were observed in the mucosa of the cecum, ascending colon, transverse colon, and descending colon, with a segmental distribution. These ulcers were coated with white moss-like deposits and exhibited nodular hyperplasia at their margins. There was no narrowing of the intestinal lumen, and the mucosa between the lesions appeared normal. Additionally, scattered aphthous ulcers or erosions were visible in the mucosa of the sigmoid colon and rectum.

**Figure 2:**
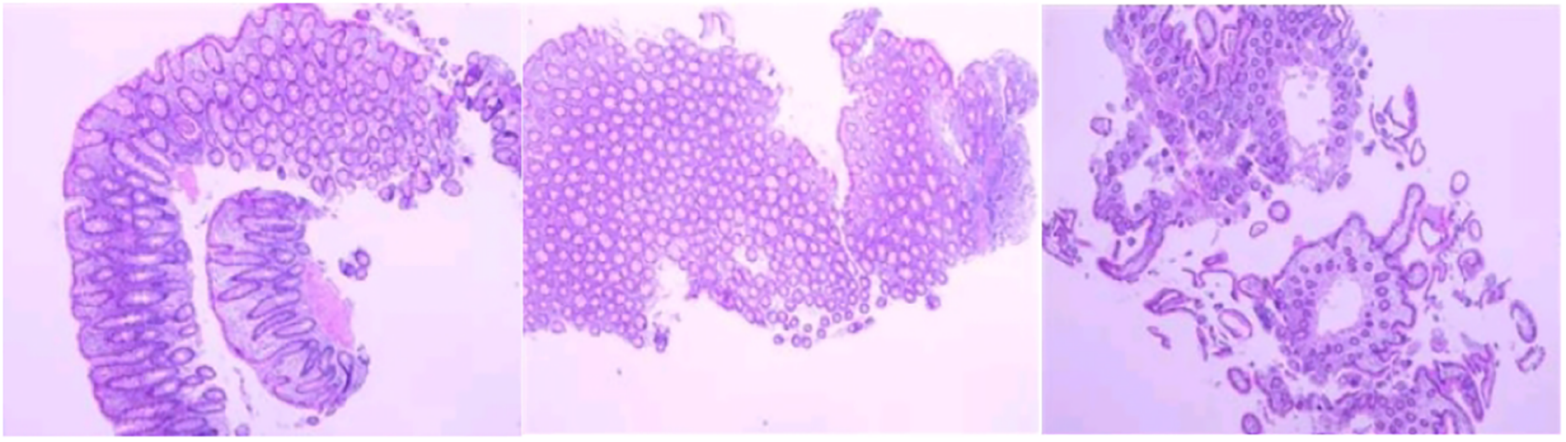
Biopsy sample analysis revealed varying degrees of active enteritis, including severe active inflammation of the descending colon, exhibiting erosion and the development of granulation tissue. No granuloma formation was observed in any of the samples (hematoxylin and eosin staining).

Magnetic resonance enterography (MRE) plain scans and enhanced scans revealed multiple, segmental, mildly increased wall thicknesses of the ileocecal, ascending, transverse, and descending colon, indicating the possibility of CD. The magnetization transfer ratio (MTR) in the intestinal wall of the descending colon was suggestive of a moderate degree of fibrosis (7–11). No active anal fistula was discernibly identified on perianal MRI. In addition, bilateral sacroiliitis was observed on MRE, which might be an extraintestinal manifestation or complication of CD. Subsequent ileo-colonoscopy revealed multiple segmental ulcerations that tended to be longitudinal (with diameters greater than 5 mm) in the colon, and intestinal skip lesions (Figure 1). The results of the biopsy sample analysis are shown in Figure 2. Histopathological examination revealed crypt distortion, crypt atrophy, and neutrophil infiltration, along with destruction of the epithelial layer. There was a significant presence of lymphocytes and plasma cells within the lamina propria. The descending colon showed the most severe involvement. Furthermore, immunohistochemistry for cytomegalovirus (CMV) and *in situ* hybridization for Epstein-Barr virus-encoded small RNA (EBER) were negative. Gastroscopy revealed congestion and edema in the gastric antrum mucosa. Ultimately, the young patient was diagnosed with CD on the basis of a combination of symptoms and radiological, ileo-colonoscopy, and histological findings. We reviewed his previous gene report and were surprised to find that, beyond the existing *LMX1B* mutation, the patient carried a heterozygous mutation in the *ATG2A* gene (chr11:64673862, c.3127G>A, p.1043, P>S), a risk factor for CD with granuloma (12).

Considering his short stature and history of extended steroid and immunosuppressant utilization, we opted not to increase the prednisone dosage for remission induction, owing to the potential adverse effects on his growth and the heightened risk of infection. On the day of his CD diagnosis, treatment with oral mesalazine was initiated. Remarkably, just 11 days later, the patient experienced significant improvement in clinical symptoms. During this period, his NS remained in remission without modification of low-dose prednisone (0.125 mg/kg/day) or tacrolimus (0.0375 mg/kg/day). A repeat endoscopy conducted 4 months later revealed near-complete resolution of the ulcers and considerable mucosal healing. Unfortunately, with the reconstitution of B cells, the patient developed massive proteinuria, necessitating an adjustment of the prednisone and tacrolimus doses to manage his NS. The prednisone dosage was adjusted to 1.5 mg/kg/day, and the tacrolimus dosage was modified to 0.05 mg/kg/day. One week later, his urine protein test results were negative. The patient's NS remained in remission for the following 4 months.

## Discussion

Rituximab (RTX), a chimeric anti-CD20 monoclonal antibody, is an important treatment for children with refractory nephrotic syndrome (NS), and has achieved favorable clinical outcomes (13). RTX can significantly reduce the rate of NS recurrence, prolong the remission period, and minimize the toxicity of steroids and immunosuppressants (14). However, the drug effect is not permanent, and most children require repeat administrations of RTX. Furthermore, the long-term safety profile of repeated RTX treatments remains uncertain (2). According to a recent retrospective study, during repeated RTX treatments, 8.0%, 6.0%, and 2.0% of pediatric patients developed hypogammaglobulinemia, severe infection, and severe neutropenia, respectively (6). However, reports of RTX-induced inflammatory bowel disease (IBD) in NS are rare at present. To our knowledge, four cases of RTX-induced IBD in NS patients have been reported and coincidentally, all patients were children (Table 2). Our case stands as the fifth instance to be documented in this realm, yet it is the first occurrence in a patient with an *ATG2A* mutation.

**Table 2:** Rituximab-induced inflammatory bowel disease in pediatric patients with nephrotic syndrome.

No.	Age(sex)	Type of IBD	Presentation of IBD	Total RTX doses	RTX regimen	Management of IBD	Renal biopsy finding	Outcome of IBD	Genetic test results
1 (15)	4 years (male)	UC	Abdominal pain, bloody diarrhea, buccal mucosa ulcers, intermittent fever	4	4-week course (375 mg/m^2^/dose per week)	Steroid, azathioprine	MCD	Rapid resolution following prednisolone (2 mg/kg/day); relapse following steroid discontinuation and complete endoscopic resolution on 7th month with steroid and azathioprine	NA
2 (16)	10 years (male)	UC	Abdominal pain, diarrhea with mucus, foul smell, and blood	6	Initial 4-week course (375 mg/m^2^/dose per week), two more doses within 1-year period	Steroid, azathioprine	MCD	Clinical remission within 2 weeks and endoscopic improvement after 6 months	NA
3 (17)	15 years (female)	CD	Abdominal pain, watery stools, weight loss	4	Initial dose of 375 mg/m^2^, three more doses within a 2-year period	Infliximab	NA	No recurrence of symptoms following infliximab therapy	NA
4 (18)	14 years (female)	CD	Prolonged low-grade fever, abdominal pain, frequent diarrhea	2	2 doses with a 12- month interval	Ustekinumab	NA	Clinical remission within 1 month and endoscopic improvement after 3 months	NA
5 (our patient)	16 years (male)	CD	Transient low-grade fever, abdominal pain, hematochezia, watery stools, multiple oral ulcers, weight loss	10	Initial dose of 375 mg/m^2^, nine more doses within a 5-year period	Mesalazine	FSGS (tip)	Clinical remission within 2 weeks and endoscopic improvement after 4 months	*LMX1B* gene (chr9:129376748, c.20C>G, p.7, P>R); *ATG2A* gene (chr11:64673862, c.3127G>A, p.1043, P>S)

IBD, inflammatory bowel disease; CD, Crohn's disease; RTX, rituximab; NA, not applicable; MCD, minimal change disease; FSGS, focal segmental glomerulosclerosis.

Our patient did not have any known environmental or familial risk factors for Crohn's disease (CD) (19). There is not enough evidence to link NS to the development of IBD to date. Conversely, RTX is a prominent factor in the onset of IBD. A population-based retrospective cohort study revealed that patients treated with RTX had an almost sevenfold increased risk of developing IBD compared with the general population, and the incidence rate of IBD among RTX-treated patients was 202 cases per 100,000 person-years (20). Our patient has received ten administrations of RTX treatment during the past 5 years, and his clinical symptoms occurred 6 months after the last RTX dose. A review of the four previous case reports revealed that the IBD symptoms of all patients developed after multiple doses of RTX, ranging from 2 to 6 (Table 2). In addition, patients who developed ulcerative colitis (UC) appeared to receive a higher initial dose of RTX than those who developed CD. Therefore, the patient's CD is considered an adverse effect (AE) of the multiple doses of RTX.

The mechanisms underlying RTX-induced IBD development have not yet been clarified. In a mouse model, intestinal injury was more severe in CD19-deficient mice than in wild-type mice (21). According to previous case reports on RTX-induced IBD, CD20+ lymphocytes are absent from the patients' gastrointestinal mucosa at CD symptom onset, but the population of T lymphocytes markedly increases throughout the course of CD (16, 22, 23). B-cell depletion may lead to impaired regulation of CD4 T-cell activity, impaired clearance of apoptotic cells in the gastrointestinal tract, and a lack of control of circulating self-antigens (24). This may be one of the mechanisms underlying RTX-induced IBD development. In addition, work in animal models indicates that, compared with that in the control group, the expression of claudin in the intestinal tissue of RTX-treated mice and the number of *Limosilactobacillus reuteri* (*L. reuteri*) in the intestines were significantly decreased (25). Claudin is a tight junction transmembrane protein and closely correlated with intestinal barrier function. L. reuteri reduces inflammatory reactions and ameliorates colitis (26). Therefore, we can infer that impaired intestinal barrier function and gut microbiota dysregulation are involved in the pathophysiology of RTX-induced IBD.

Notably, our patient had a mutation in the *ATG2A* gene. *ATG2A* is a human homolog of Atg2 found in yeasts and is an essential core member of the autophagy machinery (27). Autophagy is a catabolic process that results in the lysosome-mediated recycling of organelles and protein aggregates, and the destruction of intracellular pathogens (28). Autophagy dysregulation has been implicated in several diseases, including cancer, neurodegenerative disorders, infections, autoimmune diseases, metabolic disorders, and CD (29). A cohort study of surgically treated CD patients revealed that the presence of high risk variants of the autophagy gene *ATG2A* represents a risk factor for CD with granulomas (12). In animal models, targeted inhibition of autophagy via Atg2 RNA interference (RNAi) significantly disrupted the signaling pathways associated with mitosis and resulted in a reduction in the number of intestinal stem cells within the midgut (30). Therefore, changes in autophagy related to the *ATG2A* gene variant are likely an additional mechanism contributing to the development of IBD. Our case report provides clinical evidence supporting the relationship between the *ATG2A* gene variant and IBD.

Diarrhea and abdominal pain are the cardinal symptoms reported by patients with RTX-induced IBD (Table 2). Other symptoms include fatigue, hematochezia, weight loss, and fever, among others. Moreover, extraintestinal manifestations (EIMs) and complications of RTX-induced IBD, such as aphthous stomatitis and arthritis, should not be ignored. These EIMs may be present even before gastrointestinal symptoms appear, and are linked to intestinal disease activity (19). Our patient's first symptom was back pain, which can be explained as the clinical feature of his bilateral sacroiliitis shown on MRE. With the discontinuation of RTX and the amelioration of intestinal inflammation, the patient's oral ulcers and back pain markedly improved.

The therapeutic goal in the management of CD is mucosal healing (defined as restitution of the intestinal lining and the regression or disappearance of endoscopic lesions), which is associated with improved short-term outcomes such as a reduced risk of relapse, decreased hospitalization rates, steroid-free remission in follow-up examinations and increased remission-free intervals (31). Mesalazine treatment achieved both symptomatic remission and endoscopic remission. Prednisone and tacrolimus may also play a role in this process. At present, in addition to the discontinuation of RTX, conventional nonbiologic therapies (such as corticosteroids, mesalazine and immunosuppressants) and biologic therapies (such as infliximab and ustekinumab) have shown efficacy in treating RTX-induced IBD. In our patient, NS recurred 1 year after the patient discontinued RTX. Restarting RTX poses a significant risk of triggering a recurrence of CD, which complicates its management. Thus, maintaining a balance between the potential benefits and harms of RTX presents a considerable challenge in this patient's treatment plan. In the case reported by Morita et al. (17), infliximab (an anti-TNF-α inhibitor) can extend the remission period of NS apart from achieving the remission of CD, despite discontinuation of RTX treatment and an increase in CD19 expression. A recent study revealed that TNF-α levels were significantly elevated during the active phase in children with NS compared with healthy controls. While these levels decreased during remission, they remained significantly higher than those in the healthy control group (32). Therefore, infliximab may be an effective option for simultaneously managing CD and reducing the recurrence of NS. However, it remains imperative to substantiate this possibility with a significant amount of clinical data.

In conclusion, the possibility of RTX-induced IBD should be considered in the case of diarrhea and abdominal pain after multiple doses of RTX treatment in children with NS. Hereditary susceptibility may be a risk factor. It is paramount to promptly perform gastrointestinal endoscopy. Additionally, there is a pressing demand to identify effective strategies that can both manage RTX-induced IBD and reduce the recurrence of NS in patients with NS.

## Data Availability

The original contributions presented in the study are included in the article/Supplementary Material, further inquiries can be directed to the corresponding author.
