# Supplementary material for: Crohn's disease after multiple doses of rituximab treatment in a child with refractory nephrotic syndrome and an ATG2A mutation: a case report
Source: Front Pediatr. 2024 Nov 20;12:1464757. doi: 10.3389/fped.2024.1464757 (PMC11614595; doi:10.3389/fped.2024.1464757)
Supplement: Supplementary file 1 [file Datasheet1.docx]

Supplementary Material


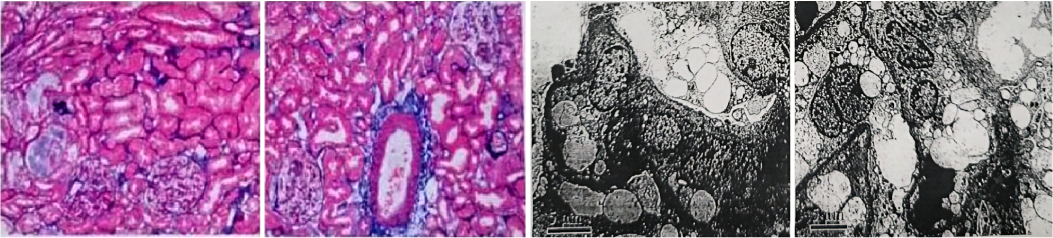


**Supplementary Figure 1.** Light and electron microscopic findings for the patient. Light microscopy revealed segmental sclerosis in 4 out of 15 glomeruli, predominantly located at the urinary pole, along with vacuolar and granular degeneration of the renal tubular epithelium, as well as focal atrophy. Additionally, small focal infiltrations of foamy cells accompanied by fibrosis were observed in the renal interstitium. Electron microscopy revealed extensive fusion of the epithelial foot processes without any deposition of electron-dense material.


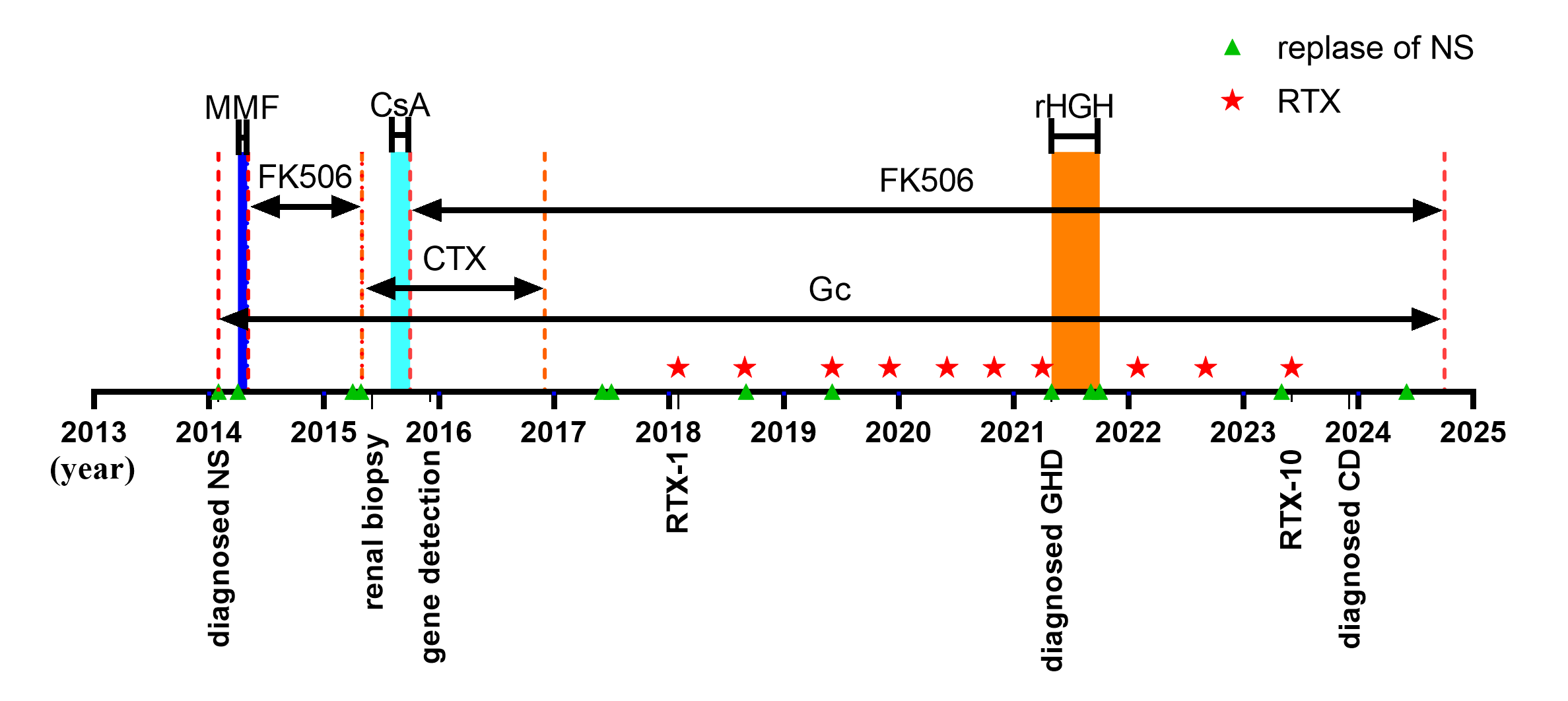


**Supplementary Figure 2.** Timeline for this patient. The timeline illustrates the progression of the patient's disease and outlines the major medical treatments administered. Green triangles represent nodes of nephrotic syndrome (NS) recurrence, while red pentagonal stars indicate the impact nodes of rituximab (RTX). NS: nephrotic syndrome; FSGS: focal segmental glomerulosclerosis; RTX: rituximab; GHD: growth hormone deficiency; CD: Crohn’s disease ; MMF: mycophenolate mofetil; CTX: cyclophosphamide; FK506: tacrolimus; CsA: ciclosporin A; rHGH: recombinant human growth hormone; Gc: glucocorticoid.
